# Association between serum homocysteine and sarcopenia among hospitalized older Chinese adults: a cross-sectional study

**DOI:** 10.1186/s12877-022-03632-0

**Published:** 2022-11-24

**Authors:** Bing Lu, Lingyu Shen, Haiqiong Zhu, Ling Xi, Wei Wang, Xiaojun Ouyang

**Affiliations:** 1 Department of Geriatrics, Geriatric Hospital of Nanjing Medical University, 65 Jiangsu Road, Nanjing, 210024 China; 2 Chronic Disease and Health Management Research Center, Geriatric Hospital of Nanjing Medical University, 65 Jiangsu Road, Nanjing, 210024 China

**Keywords:** Sarcopenia, Homocysteine, Hyperhomocysteinemia, Older adults

## Abstract

**Objective:**

Hyperhomocysteinemia (HHcy) is considered to increase the risk of sarcopenia (*S*) and remains controversial. In this study, we aimed to investigate the prevalence of *S* among older Chinese adults and explore whether homocysteine (Hcy) was independently associated with *S*.

**Methods:**

This cross-sectional study was performed among older adults hospitalized in the Geriatric Hospital of Nanjing Medical University between June 2017 and December 2021. We measured all participants’ serum Hcy levels, hand grip strength, gait speed and appendicular skeletal muscle index(ASMI) using bioelectrical impedance analysis (BIA). *S* was defined based on the criteria of the Asian Working Group for Sarcopenia 2 (AWGS2), which included muscle mass (ASMI< 7.0 kg/m^2^ for men and ASMI< 5.7 kg/m^2^ for women by BIA) and low muscle strength (handgrip strength < 28 kg for men and < 18 kg for women), and/or gait speed < 1.0 m/s. HHcy defined as Hcy ≥10 μmol/L. The strength of the association between Hcy and the risk of *S* was analyzed by multivariate logistic regression using three models that adjusted for possible confounding variables to calculate the odds ratios (ORs) and 95% confidence intervals (CIs).

**Results:**

Among the 441 subjects, 161 (36.5%) were diagnosed with *S*, and 343 (77.8%) were diagnosed with HHcy. A significant association was detected between *S* and serum Hcy per 1-μmol/L increase after adjustment for age, gender, education, smoking, body mass index (BMI), Mini Nutritional Assessment Short Form (MNA-SF), alanine aminotransferase (ALT), C-reactive protein (CRP), hemoglobin (Hb), albumin (ALB), diabetes, kidney disease, and statin use (OR = 1.07, 95% CI = 1.03–1.12, *P* = 0.002). The OR for *S* in the HHcy group (≥10 μmol/L) was nearly 5-fold that in the normal Hcy group (OR 4.96, 95% CI 2.67–9.24, *P* < 0.001). In a gender-based subgroup analysis that adjusted for age, education, smoking, BMI, MNA-SF, ALT, CRP, Hb, and ALB, female subjects with HHcy had an increased risk of *S* (OR 10.35, 95% CI 2.84–37.68, *P* < 0.001).

**Conclusions:**

Our results demonstrated that elevated Hcy levels have an independent association with *S* in older adults. This suggests that the downward adjustment of HHcy (cutoff value < 10 μmol/l) might decrease the risk of *S*.

**Supplementary Information:**

The online version contains supplementary material available at 10.1186/s12877-022-03632-0.

## Background

Sarcopenia (*S*) is an age-related syndrome characterized by decreased muscle mass and muscle strength associated with an increased risk of adverse outcomes [1], including falls and fractures, impairment in personal care, physical disability and mortality [1–3]. There are no effective pharmacological treatments for *S*, which means it is becoming a global health problem. Identifying the risk factors for *S* as soon as possible and conducting timely intervention may delay its progression [1–3].

Recently, it has been found that homocysteine (Hcy) has a certain correlation with *S* [4, 5]. As an inflammatory indicator of vascular endothelial injury and neurodegeneration, hyperhomocysteinemia (HHcy, serum homocysteine concentration ≥ 10 μmol/L) can increase the risk of cardiovascular organ damage, ischemic stroke, and Parkinson’s syndrome [6–11]. Veeranki et al. found that the HHcy-induced decrease in satellite cell proliferation involved excessive oxidative stress, and abnormal activation of p38 mitogen-activated protein kinase (MAPK) signaling led to decreased muscle regeneration based on the findings of rodent models [4].

Yamada Y et al. [5] conducted a cross-sectional study among memory clinic outpatients and demonstrated a negative association between Hcy and grip strength after adjusting for cognitive function, but this association was not universally observed in other studies. A systematic review analyzed for the first time the association of increased Hcy levels with *S* and its components, including muscle mass, muscle strength, and physical performance [12]. However, after adjustment for potential covariates, muscle strength and physical performance showed no significant associations with Hcy levels, the results were conflicting, and the relationship remains controversial. De Giuseppe and colleagues [12] pointed out that due to differences in genetics and lifestyles, the process of population aging in different regions is not consistent, and further studies should be performed to reveal the heterogeneity between “older adults”.

We aimed to investigate the prevalence of *S* among older Chinese adults and explore whether Hcy was independently associated with *S*. In addition, we evaluated the relationship between HHcy and *S* by subgroup analysis for age and sex.

## Methods

### Participants and study design

**Fig. 1:**
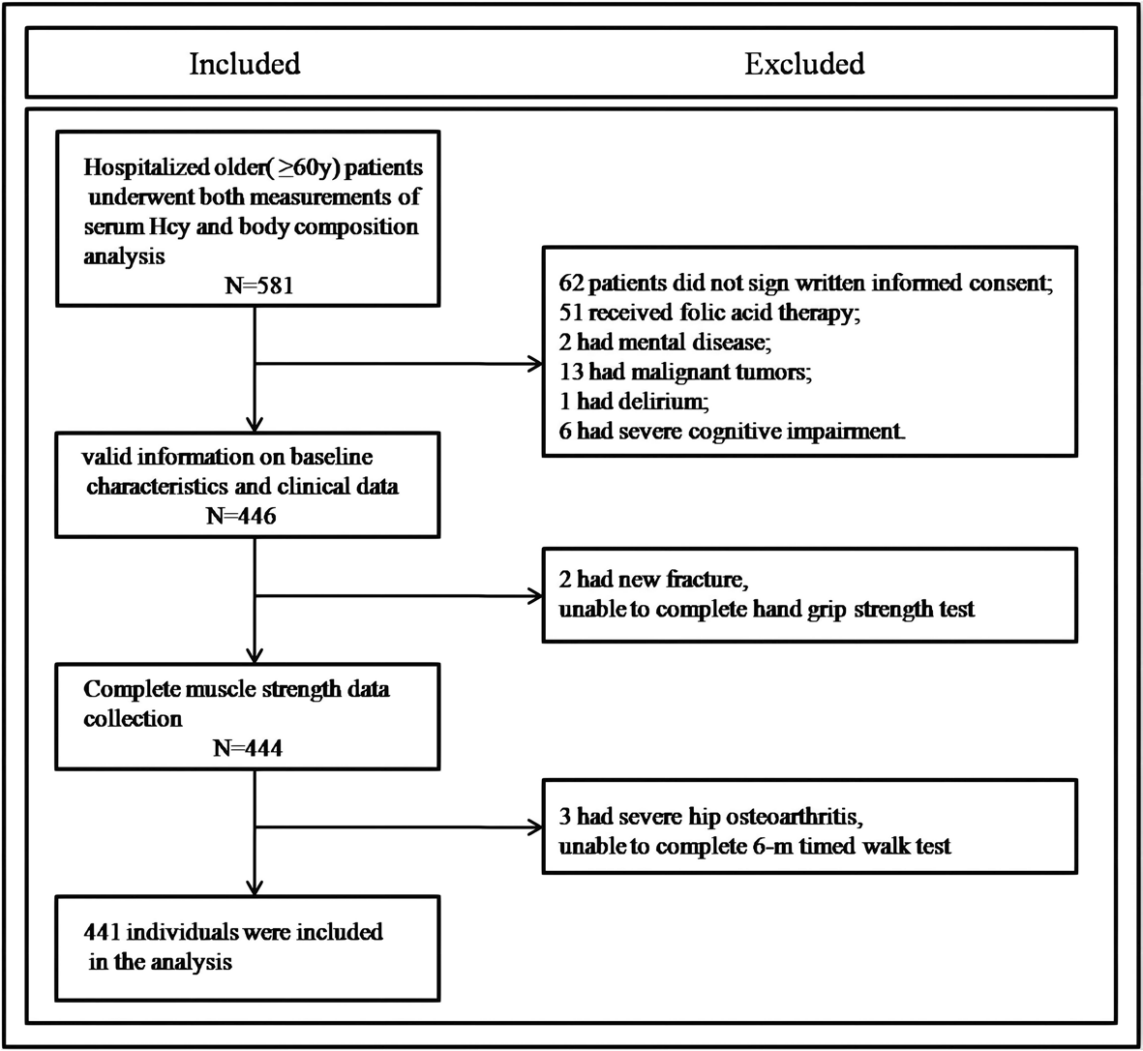
Flowchart of the participant selection process

Patients aged 60 years and older hospitalized at the Geriatric Hospital of Nanjing Medical University between June 2017 and December 2021 were enrolled in this cross-sectional study. This study was approved by the Ethics Committee of Jiangsu Province Geriatrics Institute, and written informed consent was obtained from each subject before enrollment. We excluded participants with any of the following: folic acid therapy; aphasia, delirium or severe cognitive impairment history; serious hematological diseases or malignant tumors; severe knee or hip osteoarthritis, lumbar spinal stenosis, or new fracture; history of mental illness; cardiac pacemaker implants; contraindications that meant that bioelectrical impedance analysis (BIA) could not be performed; and incomplete scale and evaluation data. Ultimately, according to the inclusion and exclusion criteria, 441 individuals were included in the analysis to determine the association between Hcy and *S*. The study flowchart is shown in Fig. 1.

### Sample size estimation

This cross-sectional study evaluated the prevalence of *S* among older Chinese adults. According to the report of the Asian Working Group for Sarcopenia 2 (AWGS2), the estimated prevalence of *S* ranges from 5.5 to 25.7% in Asia [13]. Based on the AWGS2 report, the prevalence of *S* was expected to be 25%. Two-sided tests were conducted with 5% precision and a 95% confidence level using the following sample size formula:

$$n=\left(\frac{Z_{1-\alpha/2}}\delta\right)^2\times p\times\left(1-p\right).$$

The size of the sample was computed to be 288, where Z1-α/2 is the statistic for the level of confidence, δ is the precision level, and *P* is the expected prevalence. Considering the 20% missing data, at least 360 cases had to be included in this study. This was a conservative estimate because the prevalence was expected to be higher among hospitalized older adults. Our study ultimately included 441 inpatients, so the sample size was sufficient.

### Body composition and nutritional status

Body composition was assessed by eight-electrode multifrequency BIA (Inbody S10, Korea). Adhesive electrodes were attached to the dorsum of both hands and legs with participants in the supine position. The sum of the skeletal muscle mass (SMM) of the arms and legs was estimated with the Janssen equation [14], and the appendicular skeletal muscle index (ASMI) was calculated as the appendicular SMM/height^2^(kg/m^2^). The Mini Nutritional Assessment Short Form (MNA-SF) was used as a surrogate for nutritional status [15]. The MNA-SF includes six items: food intake, weight loss, mobility, psychological stress, cognitive status and body mass index (BMI) [15]. Subjects were classified as malnourished (score ≤ 7), at risk of malnutrition (score 8–11) or well nourished (score ≥ 12) [15]. BIA and the MNA-SF were performed by one trained and experienced nutritionist.

### Data collection

Two trained nurses collected the demographic characteristics and clinical data of all subjects. Prior to the survey, standardized training was provided to all personnel in accordance with standard methods. Demographic characteristics were obtained from a questionnaire completed after enrollment and included age, gender, BMI, highest educational attainment, smoking status, alcohol intake, and personal medical history, including medication history, chronic diseases, and nutritional status. Blood samples were collected in the morning after a 12-hour overnight fast, and clinical indicators such as fasting blood glucose, hemoglobin A1c (HbA1c), lipid profiles, uric acid (UA), blood urea nitrogen (BUN), serum creatinine (Scr), alanine aminotransferase (ALT), aspartate aminotransferase (AST), thyroid-stimulating hormone (TSH), hemoglobin (Hb), albumin (ALB), and C-reactive protein (CRP) were measured in the hospital clinical laboratory [16].

### Diagnostic criteria and related measurements of *S*

According to the AWGS criteria in 2019, *S* was defined as age-related loss of muscle mass (ASMI< 7.0 kg/m^2^ for men and ASMI< 5.7 kg/m^2^ for women by BIA) plus low muscle strength (handgrip strength < 28 kg for men and < 18 kg for women) and/or low physical performance (gait speed < 1.0 m/s) [13].

Hand grip strength was assessed by a manual spring-type dynamometer. The patient was asked to test the grip strength twice on both hands, and the maximum grip strength was used for the analysis [13]. Gait speed was calculated by measuring the time to take a 6-m walk at a normal pace and expressed as meters per second (m/s) [13]. The measurement of the ASMI was depicted in Section 2.3 (body composition).

We used a nonelastic tape measure to measure the maximum circumference values of both calves and recorded the maximum value as the calf circumference [13].

The measurements of handgrip strength, gait speed and calf circumference were all performed by two trained and experienced nurses.

### Definitions

HHcy was defined as a plasma total Hcy concentration ≥ 10 μmol/L [5], which was measured by the enzymatic cycling method using a Hitachi 7020 automatic analyzer.

The educational level of the patient was classified into four groups: primary school or below, middle school, high school, and university or above. Current drinking status was defined as an alcohol consumption of ≥8 g per week [17]. Smoking in the past 6 months was defined as current smoking. BMI was calculated as weight (kg)/height (m)^2^. Subjects were divided into four categories: BMI < 18.5, 18.5 ≤ BMI < 24.0, 24.0 ≤ BMI < 28.0, and BMI ≥ 28.0.

### Statistical analysis

Data with a nonnormal distribution are expressed as M (P25, P75), continuous variables are expressed as the means ± standard deviations (SDs), and categorical data are expressed as percentages. The Wilcoxon rank sum test was used for comparisons between the two nonnormal distribution groups. Differences in continuous variables between two groups were estimated by the unpaired Student’s *t test* or Mann–Whitney *U* test according to whether the data fit a normal Gaussian distribution. A chi-square test was used to compare the differences between two categorical variables.

Multivariate logistic regression analyses were carried out to assess the strength of the association between Hcy and *S* using three models that adjusted for possible confounding variables by adjusted odds ratios (ORs) with 95% confidence intervals (CIs). We performed multicollinearity analysis, and the factors that were expected to have close relationships were excluded through a variance inflation factor (VIF) > 10. Based on clinical characteristics, variables with *P* < 0.05 in the univariate analysis were entered into the first model (Model 1), and factors associated with *S* from previous studies [18–20] were entered into Model 2 and Model 3. Model 1 was adjusted for age, sex, education, smoking, BMI, MNA-SF, ALT, CRP, Hb and ALB. The second model (Model 2) was additionally adjusted for chronic diseases such as diabetes mellitus and renal disease. Model 3 was additionally adjusted for medicine use, such as statin use. We introduced Hcy levels as either categorical (normal Hcy vs. HHcy) or continuous (increment per standard deviation in Hcy) variables in multivariable logistic regression analysis. Multivariable logistic regression was repeated by using Model 1 for subgroup analyses by age (< 85 years vs. ≥ 85 years) and sex (male vs. female).

All statistical analyses were performed using the Statistical Package for the Social Sciences (SPSS) version 22.0 software (SPSS Inc., Chicago, USA), and a two-tailed *P value* < 0.05 was considered statistically significant.

## Results

### Demographic and clinical characteristics

Among the 441 participants, 262 (59.4%) were male, and 161 (36.5%) met the diagnostic criteria for *S*. The prevalence of HHcy was 77.8%. The mean age of all participants was 81.3 ± 9.5 years*.* There were significant differences in age, sex, education, smoking, BMI, MNA-SF, ALT, CRP, Hb, ALB and Hcy between the two groups (all *P* < 0.05, Table 1).

**Table 1:** Demographic and clinical characteristics of subjects

Variable	Total(441)	Non-sarcopenia(280)	sarcopenia(161)	*t*/*χ*^*2*^/*Z*	*p* value
Age(year)	81.3 ± 9.5	79.6 ± 10.0	84.3 ± 7.7	5.458	< 0.001
Gender(male)	262 (59.4)	155 (55.4)	107 (66.5)	5.225	0.022
Education					
Primary or below	37 (8.4)	14 (5.0)	23 (14.3)	3.274	0.001
Middle school	75 (17.0)	43 (15.4)	32 (19.9)		
High school	138 (31.3)	90 (32.1)	48 (29.8)		
University or above	191 (43.3)	133 (47.5)	58 (36.0)		
Smoking	109 (24.7)	58 (20.7)	51 (31.7)	6.602	0.010
Drinking	129 (29.3)	81 (28.9)	48 (29.8)	0.039	0.844
BMI(kg/m^2^)					
BMI < 18.5	11 (2.5)	4 (1.4)	7 (4.3)	6.459	< 0.001
18.5 ≤ BMI < 24.0	176 (39.9)	82 (29.3)	94 (58.4)		
24.0 ≤ BMI < 28.0	176 (39.9)	131 (46.8)	45 (28.0)		
BMI ≥ 28.0	78 (17.7)	63 (22.5)	15 (9.3)		
Statin use	338 (76.6)	216 (77.1)	122 (75.8)	0.107	0.744
Diabetes	197 (44.7)	123 (43.9)	74 (46.0)	0.171	0.679
Hypertension	286 (64.9)	184 (65.7)	102 (63.4)	0.250	0.617
Kidney disease	23 (5.2)	15 (5.4)	8 (5.0)	0.031	0.860
MNA-SF					
Well-nourished	343 (77.8)	249 (88.9)	94 (58.4)	7.445	< 0.001
Malnutrition risk	89 (20.2)	29 (10.4)	60 (37.3)		
Malnutrition	9 (2.0)	2 (0.7)	7 (4.3)		
Systolic BP (mmHg)	134.7 ± 16.0	134.4 ± 15.4	135.1 ± 17.1	0.448	0.654
Diastolic BP (mmHg)	72.7 ± 9.5	72.9 ± 9.1	72.3 ± 10.1	0.734	0.463
ALT(U/L)	15.0 (12.0,22.0)	16.0 (12.0,23.0)	14.0 (11.0,21.0)	2.184	0.029
AST(U/L)	18.0 (15.0,22.0)	18.0 (15.0,22.0)	18.0 (15.0,21.0)	0.560	0.575
BUN(mmol/L)	5.6 (4.6,6.9)	5.5 (4.6,6.8)	5.8 (4.7,7.3)	1.384	0.166
Scr(umol/L)	76.0 (64.0,91.5)	75.0 (63.9,87.4)	78.0 (64.2,97.7)	0.995	0.320
SUA(mg/dL)	324.0 (265.0,390.5)	325.0 (270.5390.5)	322.0 (261.0,390.5)	0.311	0.756
TG (mmol/L)	1.1 (0.8,1.5)	1.1 (0.8,1.6)	1.0 (0.8,1.4)	1.226	0.220
LDL-C (mmol/L)	2.3 (1.7,2.8)	2.3 (1.8,2.9)	2.2 (1.7,2.8)	0.563	0.573
HDL-C (mmol/L)	1.1 (0.9,1.4)	1.1 (0.9,1.3)	1.1 (0.9,1.4)	1.354	0.176
HbA1c(%)	6.2 (5.8,6.8)	6.2 (5.8,6.7)	6.2 (5.8,6.9)	0.298	0.766
K(mmol/L)	3.9 (3.7,4.1)	3.9 (3.7,4.1)	3.9 (3.6,4.1)	0.347	0.729
CRP(mg/L)	1.3 (0.6,3.6)	1.1 (0.5,2.9)	1.7 (0.7,6.2)	2.824	0.005
TSH(uIU/ml)	2.0 (1.3,2.8)	1.9 (1.3,2.7)	2.2 (1.4,3.2)	1.494	0.135
Hcy(umol/L)	12.5 (10.3,15.5)	11.7 (9.6,14.9)	13.4 (11.6,17.3)	4.919	< 0.001
Hb(g/L)	127.6 ± 17.4	129.1 ± 17.5	124.8 ± 16.7	2.520	0.012
ALB(g/L)	38.2 ± 4.3	38.8 ± 4.1	37.4 ± 4.6	3.303	0.001
TP(g/L)	66.4 ± 6.9	66.8 ± 6.8	65.6 ± 7.1	1.763	0.079
EF(%)	65.7 (63.7,68.5)	65.8 (63.7,68.5)	65.6 (63.7,68.5)	0.385	0.701
Calf circumference(cm)	33.0 (31.0,36.0)	34.0 (32.0,36.0)	32.0 (30.0,34.0)	7.534	< 0.001
Maximum grip(kg)					
Male	26.2 ± 7.9	29.1 ± 7.8	22.1 ± 6.1	8.074	< 0.001
Female	20.6 ± 6.8	22.6 ± 6.7	15.9 ± 4.4	7.977	< 0.001
6 m pace(m/s)	0.7 ± 0.3	0.8 ± 0.3	0.6 ± 0.3	4.060	< 0.001
SMM(kg)					
Male	25.6 ± 4.6	28.2 ± 3.5	21.8 ± 3.0	15.702	< 0.001
Female	20.5 ± 4.3	22.1 ± 3.8	16.7 ± 2.7	10.808	< 0.001
ASMI(kg/m^2^)					
Male	7.3 ± 1.2	8.0 ± 1.0	6.3 ± 0.7	16.164	< 0.001
Female	6.3 ± 1.0	6.8 ± 0.8	5.2 ± 0.5	16.901	< 0.001

*BMI* body mass index, *MNA-SF* mininutritional assessment short form, *ALT* alanine transaminase, *AST* aspartate transaminase, *BUN* Blood urea nitrogen, *Scr* serum creatinine, *SUA* serum uric acid, *TG* triglyceride, *LDL-C* low-density lipoprotein cholesterol, *HDL-C* high density lipoprotein cholesterol, *HbA1c* glycosylated hemoglobin, *K* kalium, *CRP* C-reactive protein, *TSH* thyroid stimulating hormone, *Hcy* homocysteine, *Hb* hemoglobin, *ALB* albumin, *TP* total protein, *EF* ejection fraction, *SMM* skeletal muscle mass, *ASMI* appendicular skeletal muscle index

As expected, patients with *S* demonstrated lower calf circumference [32.0 (30.0, 34.0) cm vs. 34.0 (32.0, 36.0) cm, *P* < 0.001], slower gait speed (0.7 ± 0.3 m/s vs. 0.8 ± 0.3 m/s, *P* < 0.001), lower ASMI (6.3 ± 0.7 kg/m^2^ vs. 8.0 ± 1.0 kg/m^2^, 5.2 ± 0.5 kg/m^2^ vs. 6.8 ± 0.8 kg/m^2^, *P* < 0.001 for men and women, respectively) and decreased grip strength (22.1 ± 6.1 kg/m^2^ vs. 29.1 ± 7.8 kg/m^2^, 15.9 ± 4.4 kg/m^2^ vs. 22.6 ± 6.7 kg/m^2^, *P* < 0.001 for men and women, respectively). The Hcy level was significantly higher in the *S* group than in the non-*S* group [13.4 (11.6, 17.3) μmol/L vs. 11.7 (9.6, 14.9) μmol/L, *P* < 0.001].

### Multivariate analysis of the risk factors for *S*

**Fig. 2:**
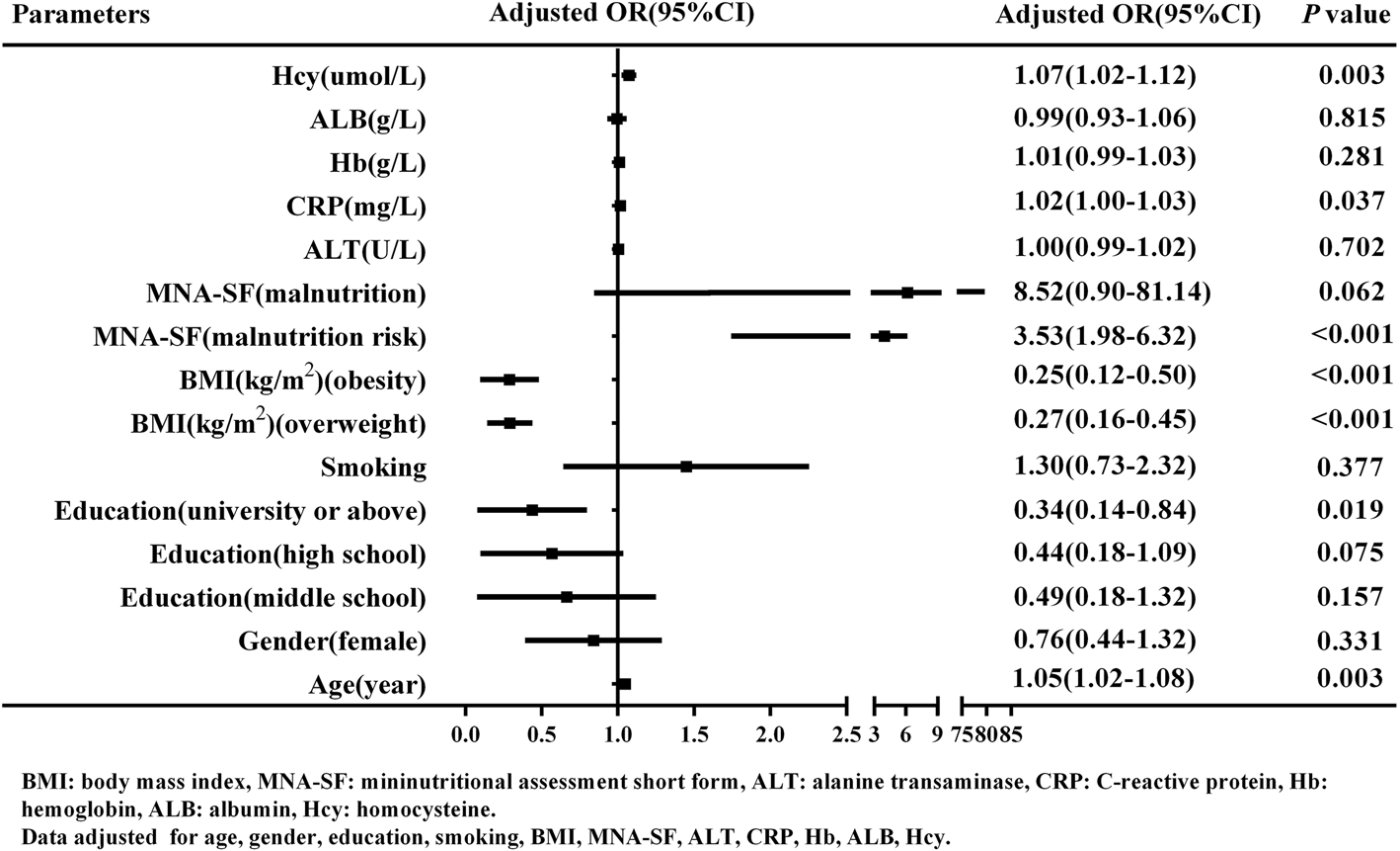
Multivariate analysis of the risk factors for *S.* The variables with *P* < 0.05 in the univariate analysis between the *S* group and the non-*S* group were introduced into the multivariate logistic regression model. After adjustment for age, gender, education, smoking, BMI, MNA-SF, ALT, CRP, Hb, and ALB, a significant positive association was detected between *S* and serum Hcy per 1-μmol/L increase (OR = 1.07, 95% CI = 1.02–1.12, *P* = 0.003). BMI (obesity and overweight) was negatively correlated with *S*. The risk of *S* was significantly decreased in the university education or above group. Moreover, the MNA-SF, CRP, and age were significantly associated with an increased risk of *S*

The variables with *P* < 0.05 in the univariate analysis between the *S* group and the non-*S* group were introduced into the multivariate logistic regression model. We conducted multicollinearity analysis on Hb, ALT, and ALB, which showed VIFs of 1.2, 1.0, and 1.2, respectively. Then, we repeated the same analysis on BMI and the MNA-SF, and the VIFs were both 1.1 (Supplementary Fig. 1, Supplementary Fig. 2). There was no multicollinearity for these variables. After adjustment for age, gender, education, smoking, BMI, MNA-SF, ALT, CRP, Hb, and ALB, as shown in Fig. 2, a significant positive association was detected between *S* and serum Hcy per 1-μmol/L increase (OR = 1.07, 95% CI = 1.02–1.12, *P* = 0.003). BMI (OR, 0.25; 95% CI, 0.12 to 0.50 for obesity and OR, 0.27; 95% CI 0.16 to 0.45 for overweight, all *P* < 0.001) was negatively correlated with *S*. The risk of *S* was significantly decreased in the university education or above group (OR 0.34, 95% CI 0.14–0.84, *P* = 0.019). Moreover, age (OR 1.05, 95% CI 1.02–1.08, P = 0.003), CRP (OR 1.02, 95% CI 1.00–1.03, *P* = 0.037) and the MNA-SF (OR, 3.53; 95% CI 1.98 to 6.32 for malnutrition risk, *P* < 0.001) were significantly associated with an increased risk of *S*.

### The association between Hcy and the risk of *S* in multivariable analysis

As presented in Table 2, there was a significant association between Hcy and *S* in crude model multivariable analysis (OR 1.09, 95% CI 1.05–1.13, *P* < 0.001). In Model 1, which adjusted for age, sex, education, smoking, BMI, MNA-SF, ALT, CRP, Hb, and ALB, there was a significant association between Hcy and S (OR 1.07, 95% CI 1.02–1.12, *P* = 0.003). Analysis using Model 2, which additionally adjusted for chronic disease (diabetes and kidney disease), showed that Hcy was associated with *S* (OR = 1.07, 95% CI 1.05–1.12, *P* = 0.002). In Model 3, which additionally adjusted for medicine use, such as statin use, elevated Hcy levels were significantly associated with an increased risk of *S* (OR = 1.07, 95% CI 1.03–1.12, *P* = 0.002).

**Table 2:** The association between homocysteine and the risk of sarcopenia in multivariable analysis

		OR(95%CI)	*p* value
Crude		1.09 (1.05–1.13)	< 0.001
Adjusted	Model 1	1.07 (1.02–1.12)	0.003
	Model 2	1.07 (1.05–1.12)	0.002
	Model 3	1.07 (1.03–1.12)	0.002

Model 1: Data adjusted for age, gender, education, smoking, *BMI* body mass index, *MNA-SF* mininutritional assessment short form, *ALT* alanine transaminase, *CRP* C-reactive protein, *Hb* hemoglobin, *ALB* albumin

Model 2: Model 1 plus adjustment for diabetes and kidney disease

Model 3: Model 2 plus adjustment for statin use

We conducted multivariable logistic regression analysis by introducing Hcy levels as a categorical variable (normal Hcy vs. HHcy) (Table 3). It was found that HHcy increased the risk of *S* more than fourfold (all *P* < 0.001).

**Table 3:** The association between homocysteine and the risk of sarcopenia in multivariable analysis

	Crude OR(95%CI)	Adjusted OR(95%CI)		
		Model 1	Model 2	Model 3
Hcy < 10.0	1.00	1.00	1.00	1.00
Hcy ≥ 10.0	4.96 (2.67–9.24)	4.00 (1.98–8.10)	4.21 (2.06–8.60)	4.22 (2.07–8.61)
*p* value	< 0.001	< 0.001	< 0.001	< 0.001

Model 1: Data adjusted for age, gender, education, smoking, body mass index (BMI), mininutritional assessment short form (MNA-SF), alanine transaminase (ALT), C-reactive protein (CRP), hemoglobin (Hb), albumin (ALB)

Model 2: Model 1 plus adjustment for diabetes and kidney disease

Model 3: Model 2 plus adjustment for statin use

### Subgroup analysis of the risk for *S* by Hcy based on age and gender

Subgroups by age were analyzed (< 85 years vs. ≥85 years). As presented in Table 4, after adjusting for gender, education, smoking, BMI, MNA-SF, ALT, CRP, Hb and ALB, HHcy showed a significant association with an increased risk of *S* in the two groups (OR 7.84, 95% CI 2.42 to 25.46 for age ≥ 85 years and OR 2.9, 95% CI 1.1 to 6.63 for age < 85 years, *P* = 0.001 vs. *P* = 0.031, respectively). Moreover, no interaction effect between age and Hcy influenced the risk of *S* (*P* for interaction =0.39).

**Table 4:** Subgroup analysis of the risk for sarcopenia by homocysteine levels based on age

	Hcy < 10.0		Hcy ≥ 10.0		Crude		Adjusted	
	N	sarcopenia	N	sarcopenia	OR(95%CI)	*p* value	OR(95%CI)	*p* value
Age < 85	62	9 (14.5%)	155	54 (34.8%)	3.15 (1.44–6.87)	0.004	2.90 (1.10–7.63)	0.031
Age ≥ 85	36	4 (11.1%)	188	94 (50.0%)	8.00 (2.72–23.51)	< 0.001	7.84 (2.42–25.46)	0.001
*p* value of interaction						0.170		0.390

Data adjusted for gender, education, smoking, *BMI* body mass index, *MNA-SF* mininutritional assessment short form, *ALT* alanine transaminase, *CRP* C-reactive protein, *Hb* hemoglobin, *ALB* albumin

*Hcy* homocysteine

As shown in Table 5, after adjusting for age, education, smoking, BMI, MNA-SF, ALT, CRP, Hb, and ALB, HHcy significantly increased the risk of *S* in the female group (OR 10.35, 95% CI 2.84–37.68, *P* < 0.001). In addition, there was an interaction effect between sex and Hcy that influenced *S* (*P* for interaction =0.047).

**Table 5:** Subgroup analysis of the risk for sarcopenia by homocysteine levels based on gender

	Hcy < 10.0		Hcy ≥ 10.0		Crude		Adjusted	
	N	sarcopenia	N	sarcopenia	OR(95%CI)	*p* value	OR(95%CI)	*p* value
Male	37	8 (21.6%)	225	99 (44.0%)	2.85 (1.25–6.50)	0.013	1.94 (0.77–4.87)	0.158
Female	61	5 (8.2%)	118	49 (41.5%)	7.95 (2.97–21.31)	< 0.001	10.35 (2.84–37.68)	< 0.001
*p* value of interaction						0.117		0.047

Data adjusted for age, education, smoking, *BMI* body mass index, *MNA-SF* mininutritional assessment short form, *ALT* alanine transaminase, *CRP* C-reactive protein, *Hb* hemoglobin, *ALB* albumin

*Hcy* homocysteine

## Discussion

The present study showed that Hcy was independently associated with *S* among older hospitalized adults after adjustment for age, gender, education, smoking, BMI, MNA-SF, ALT, CRP, Hb, ALB, diabetes, kidney disease, and statin use. Additionally, subgroup analysis based on gender found that the association between Hcy and *S* was significant among females. This result has certain clinical importance and lays the foundation for further evaluation of the efficacy of reducing Hcy in specific populations.

In this study, we identified a 36.5% prevalence of *S* among hospitalized older adults. The prevalence of *S* varies greatly among older adults, ranging from 7% to over 50% [21, 22]. In Asia, the estimated prevalence of *S* ranges from 5.5 to 25.7%, with a male majority (5.1–21.0% in men vs. 4.1–16.3% in women) [13]. A report from the UK found that forty-four (10%) out of 432 acutely ill older patients were diagnosed with *S* (mean age 79 years) [23]. A study of 465 older Canadian adults using dual X-ray absorptiometry (DXA) showed that the prevalence of *S* was 38.9% vs. 17.8% among men and women, respectively [24]. In a primary care setting, using AWGS criteria, 58% of community-dwelling older patients with type 2 diabetes (T2D) had presarcopenia *(Pre-S)* and *S* in Singapore [25].

The prevalence of *S* was 36.5% among community-dwelling older adults in the United States (with a mean age of 70.1 years) based on data from Brown JC et al. [26]. Different cutoff points and criteria used to evaluate muscle mass may lead to different prevalence rates of *S* [1, 13, 27–30]. The definition of *S* according to EWGSOP criteria emphasizes that muscle strength is the primary indicator [1]. Compared with EWGSOP, AWGS believes that the decline in muscle strength and physical function is the result of the decline in muscle mass and has a negative impact on prognosis. Consequently, as long as muscle strength or function decreases, combined with the decline in muscle mass, *S* can be diagnosed [1, 13]. Therefore, the prevalence of *S* among older adults may depend on age, gender, race, living environment, disease status and different criteria.

Until recently, the complex etiology and pathogenesis of *S* were not fully understood [1, 13]. The probable mechanism behind the loss of muscular mass and strength caused by *S* is a disturbance of the muscular regeneration process. HHcy-mediated epigenetic changes may impair skeletal muscle function. Possible molecular mechanisms for the link between *S* and Hcy include compromised antioxidant capability, hypomethylation inflammation, inactivation of the nitric oxide synthase pathway, enhanced endoplasmic reticulum (ER) stress, and changes in cell signaling pathways of transforming growth factor (TGF-β) and GPCR (G protein-coupled receptor) [1, 27–31]. Through the aforementioned mechanisms, Hcy may increase the protein hydrolysis of muscle and reduce its regeneration ability, eventually leading to the occurrence of *S* [31].

At present, there is no consensus regarding the association of Hcy and *S*. For instance, Lee et al. [32] conducted a cross-sectional analysis of 1582 participants and showed a positive association between increased Hcy levels and *S*. In contrast, Eguchi et al. [33] found no significant association between Hcy levels and *S* in Japan. Choi JH et al. [34] conducted a cross-sectional study of 114,583 community-dwelling adults (18 to 95 years old). Although Choi JH confirmed the association between low SMM and HHcy, their study did not reveal an association between HHcy and *S*. In the current study, we clearly identified a positive association between elevated levels of Hcy and *S*. Another important finding is that the OR for the risk of *S* in the HHcy group (≥10 μmol/L) was nearly 5-fold that in the normal Hcy group. The possible reason for this is that insufficient Hcy metabolism may lead to skeletal muscle dysfunction [34, 35]. In our study population, 343 (77.8%) patients were found to have HHcy. It is widely known that elevated Hcy levels are connected with several conditions, including enzyme mutations, hormonal, nutritional, and vitamin B group levels (folate, vitamin B12 and B6), sex and age, genetics, lifestyle, chronic diseases and medications, and those factors involved in abnormal Hcy metabolism may result in HHcy [7–12, 31, 36]. Deficiencies in folate and vitamin B12 levels are the major reasons for the increase in Hcy levels [11]. Bulut and colleagues [37] reported that vitamin B12 deficiency might be related to *S* in older adults; however, they did not measure Hcy levels directly. A pilot cross-sectional study [38] indicated that levels of vitamin B12 did not significantly predict muscle strength in regression analysis. Since the evidence to date is vague, further in-depth research is necessary. In addition, compared to the non-*S* group, the *S* group patients had a higher proportion of malnutrition risk and malnutrition. Our outcomes coincide with those of previous observational studies [36, 39]. These results suggest the necessity of nutritional interventions in the *S* population*.*

In a gender-based subgroup analysis, female participants with HHcy had a significantly increased risk of *S*. The results coincide with those from Lee et al., who observed stronger associations between high levels of Hcy and *S* among women [32]. Currently, we have no explanation for such variability between the gender, which may be related to sex-specific pathophysiology [12, 40]. Carrying a hyperpolarization signal is very important for the gap junction composed of connexins to perform vasodilation, which can mediate skeletal muscle to obtain more nutrition [41, 42]. At present, it is not clear how HHcy regulates the expression of connexin, but it was reported that HHcy reduces the expression of connexin in the skeletal muscle vascular system, disturbs the conductance of vasodilatation (CVD) and tissue perfusion, and leads to increased fatigue [43]. These findings may explain why elderly individuals have higher Hcy levels and less exercise endurance. In addition, the increase in Hcy levels reduces the methylation and antioxidant capacity of the body and further aggravates the impact of age on muscle [44]. From what was discussed above, it is necessary for us to take more active measures to reduce Hcy levels in older women.

Multivariate regression analysis showed that a university or above education level was a protective factor against *S*. A possible explanation is that people with higher education levels are more willing to accept health education and have better compliance. It has been reported that smoking and a higher disease burden may increase serum homocysteine levels [45–47]. However, we did not observe a positive correlation between cigarette smoking and *S*, which may be related to the fact that this was only a single-center study, and selection bias may have occurred in the selection of patients. Regardless of further adjustment for chronic diseases or factors such as individual smoking habits and statin use, Hcy maintained a significant association with *S* in our study. These findings are similar to those of other studies from Asia [32, 34].

There are a few limitations in this study. First, it is impossible to assume causality from the association between *S* and Hcy. Second, all data were collected from the Geriatric Hospital of Nanjing Medical University. The average age of the present study population was over 81 years, and there might be selection bias due to the age of the participants. Third, information about vitamin B12 deficiency was missing in our study, which may cause bias. Fourth, although we used multifrequency BIA to distinguish extracellular fluid from intracellular fluid [48], for patients with special obesity or obvious edema, the formula for determining body composition derived from BIA has certain limitations in accuracy [49]. Therefore, our findings should be interpreted with caution.

In conclusion, this study demonstrated that elevated Hcy levels had an independent association with *S* among hospitalized older Chinese adults and that the relationship remained significant after adjustment for various confounding variables. Our findings strengthen the assumption of an association between Hcy and *S*. This suggests that the downward adjustment of Hcy (cutoff value < 10 μmol/l) might decrease the risk of *S*; therefore, a large randomized controlled trial is recommended to open new avenues for formulating more effective treatment strategies.

## Supplementary Information

**Additional file 1:** **Excel 1.** Raw data.

**Additional file 2: Fig. S1.** Multicollinearity analysis for Hb, ALT and ALB.

**Additional file 3: Fig. S2.** Multicollinearity analysis for BMI and the MNA-SF.

## Data Availability

Additional file 1: Excel 1. Raw data. S. Fig. 1. Multicollinearity analysis for Hb, ALT and ALB. S. Fig. 2. Multicollinearity analysis for BMI and the MNA-SF. The datasets used and/or analyzed during the current study are available from the corresponding author upon reasonable request. E-mail address: xiaojun_ouyang@aliyun.com

## Abbreviations

HHcy: Hyperhomocysteinemia
S: Sarcopenia
Hcy: Homocysteine
OR: Odds ratio
CI: Confidence interval
SPSS: Statistical Package for the Social Sciences
MAPK: Mitogen-activated protein kinase
BIA: Bioelectrical impedance analysis
BMI: Body mass index
HbA1c: Hemoglobin A1c
UA: Uric acid
BUN: Blood urea nitrogen
Scr: Serum creatinine
ALT: Alanine aminotransferase
AST: Aspartate aminotransferase
TSH: Thyroid-stimulating hormone
Hb: Hemoglobin
ALB: Albumin
CRP: C-reactive protein
MNA-SF: Mini Nutritional Assessment Short Form
AWGS: Asian Working Group for Sarcopenia
ASMI: Appendicular skeletal muscle index
EWGSOP: European Working Group on Sarcopenia in Older People
ER: Endoplasmic reticulum
TGF-β: Transforming growth factor β
GPCR: G protein-coupled receptor
CVD: Conductance of vasodilatation
