# Supplementary figures and images for: Association between serum homocysteine and sarcopenia among hospitalized older Chinese adults: a cross-sectional study
Source: BMC Geriatr. 2022 Nov 24;22:896. doi: 10.1186/s12877-022-03632-0 (PMC9685861; doi:10.1186/s12877-022-03632-0)

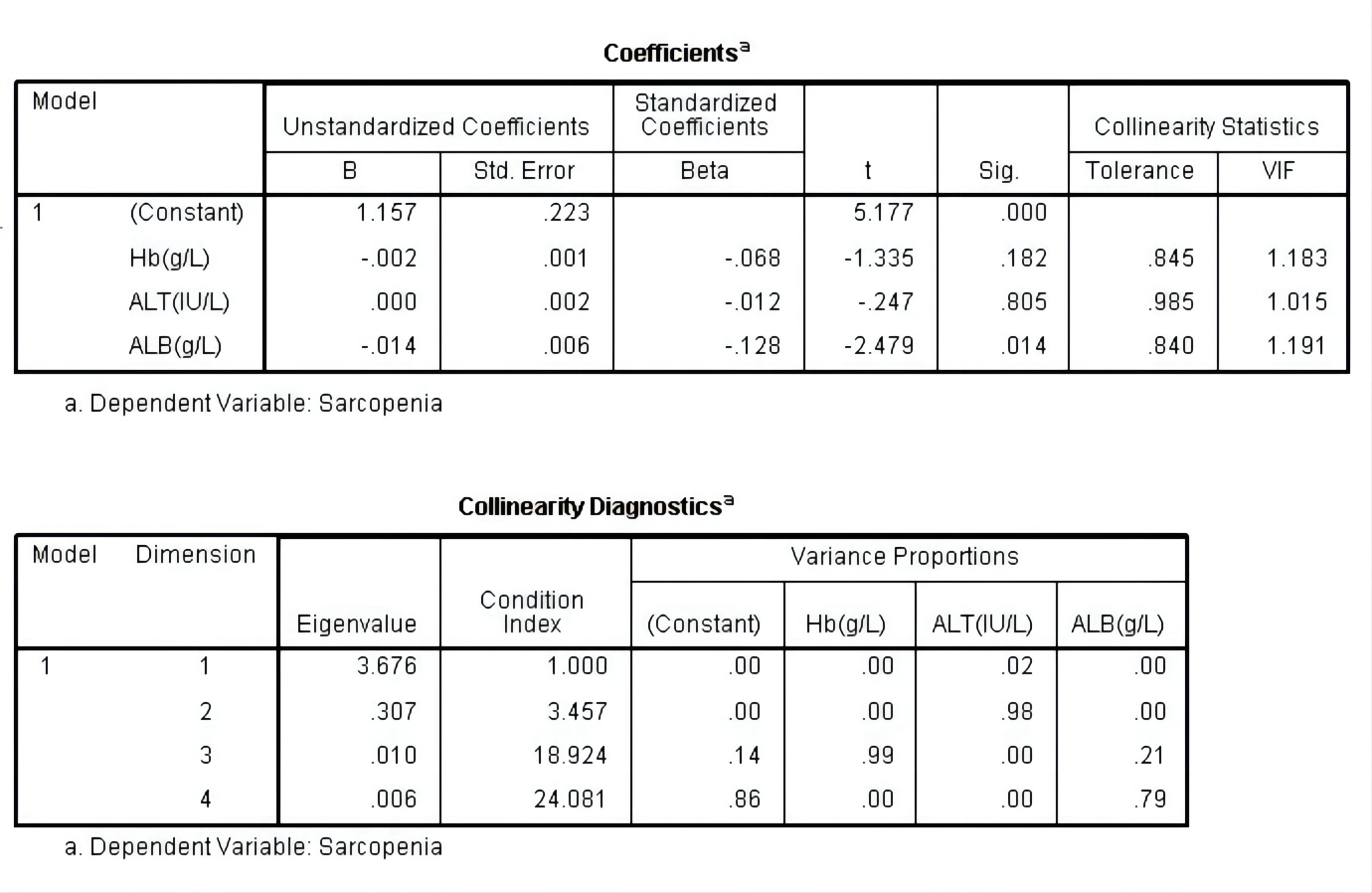

Supplement: Supplementary file 2 — Additional file 2: Fig. S1. Multicollinearity analysis for Hb, ALT and ALB. [file 12877_2022_3632_MOESM2_ESM.png]

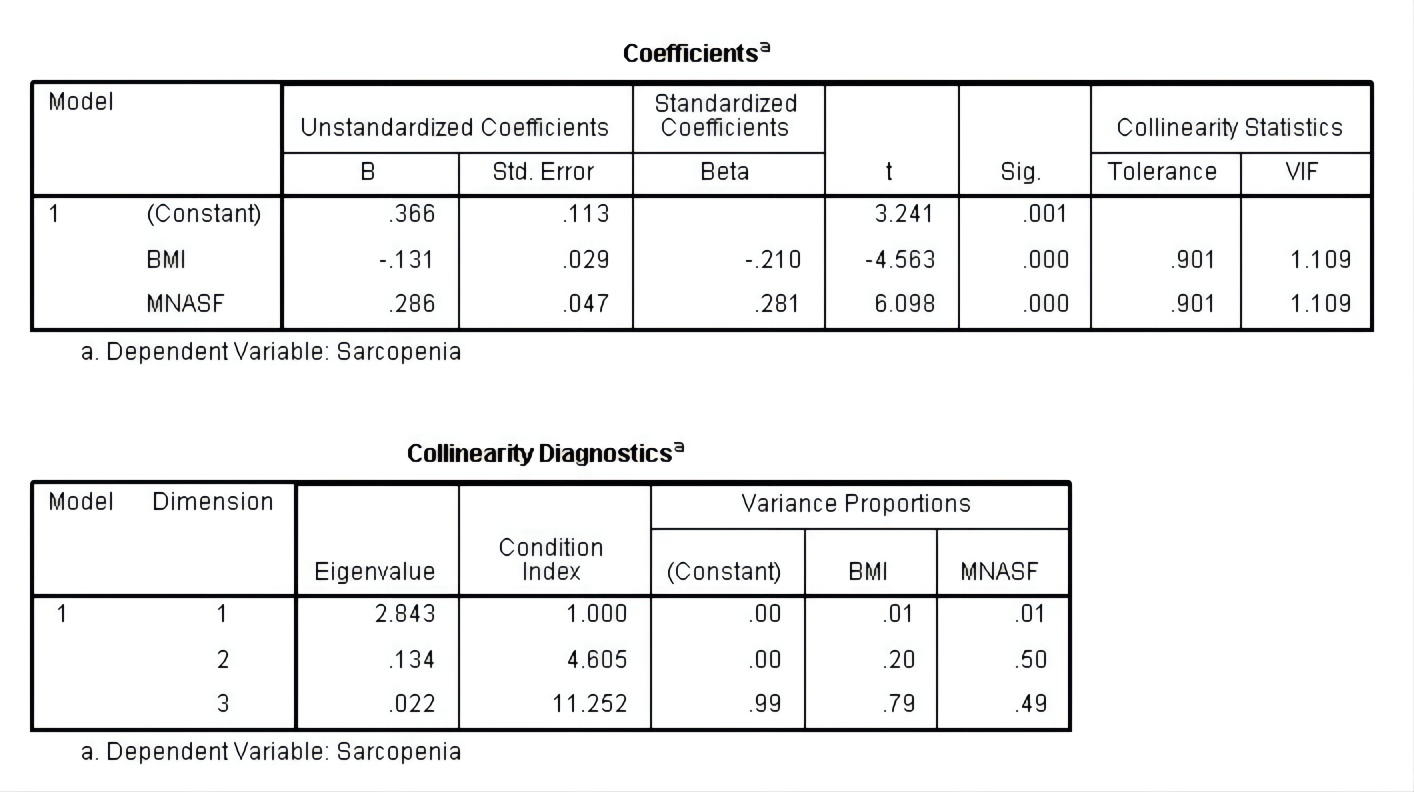

Supplement: Supplementary file 3 — Additional file 3: Fig. S2. Multicollinearity analysis for BMI and the MNA-SF. [file 12877_2022_3632_MOESM3_ESM.png]
